# Professionally successful adults with attention-deficit/hyperactivity disorder (ADHD): Compensation strategies and subjective effects of pharmacological treatment

**DOI:** 10.1590/S1980-57642009DN20100013

**Published:** 2008

**Authors:** Andre Palmini

**Affiliations:** From the Department of Internal Medicine, Division of Neurology, Faculty of Medicine, Pontificia Universidade Católica do Rio Grande do Sul (PUCRS) and Neurology Service, Hospital São Lucas, PUCRS, Porto Alegre, RS, Brazil.

**Keywords:** Neuropsychological tests, attention-deficit/hyperactivity, executive dysfunction, treatment, Testes neuropsicológicos, déficit de atenção/hiperatividade, disfunção executiva, tratamento

## Abstract

Although as a group, adult patients with ADHD have difficulties in social functioning due to inattention and executive dysfunction, some strive and succeed in living a productive, independent life.

**Purpose:**

To report on professionally successful adults with ADHD and analyze their main symptoms, compensation strategies and the subjective effect of methylphenidate on their functioning.

**Methods:**

The main symptoms of five patients with ADHD who are University educated and financially independent are reported. These patients were selected from a personally followed cohort of adults with ADHD. All were diagnosed according to DSM-IV adapted criteria (K-SADS E, version 6.0) and completed the Portuguese translated version of the ADHD adult self-reporting scale (ASRS).

**Results:**

Main reported symptoms included difficulties with attention, tendency to procrastinate and to ‘shuffle’ priorities, excessive daytime somnolence, memory difficulties and impulsiveness. Compensation strategies revolve around conscious, ‘energy demanding’ and time consuming efforts to control and circumvent symptomatic behavioral tendencies. They feel methylphenidate helps by alleviating the need to constantly apply compensation strategies for socially disabling symptoms. In sum, they achieve the same results in a more natural, less effortful fashion.

**Conclusions:**

Adults with ADHD may succeed professionally despite significant symptoms of inattention and executive dysfunction. They do so by appropriately using effortful strategies of compensation, the need for which is alleviated by the use of methylphenidate. These subjective reports require confirmation in prospective studies on larger series of patients.

## Abstract (pt)

Embora a maior parte dos pacientes adultos com TDAH apresentem dificuldades no seu funcionamento profissional, alguns conseguem ser bem sucedidos e levam uma vida produtiva e independente.

**Objetivo:**

Relatar adultos com TDAH profissionalmente bem sucedidos e analisar seus principais sintomas, suas estratégias para compensar estes sintomas e os efeitos subjetivos do metilfenidato no seu funcionamento.

**Métodos:**

São relatadas as histórias clínicas de cinco pacientes com TDAH que completaram um curso superior e são financeiramente independentes, selecionados ao acaso a partir de uma coorte de adultos com TDAH. Todos foram diagnosticados segundo critérios do DSM-IV adaptados para adultos (K-SADS E, versão 6.0) e completaram a versão adaptada para o português do adult self-report scale (ASRS) para sintomas de TDAH.

**Resultados:**

Os principais sintomas relatados incluíram dificuldades com atenção, tendência a procrastinar e ‘embaralhar’ as prioridades, sonolência diurna excessiva, dificuldades de memória e impulsividade. As estratégias de compensação utilizadas giram em torno de esforços conscientes, que demandam um alto nível de ‘energia’ e tempo, para controlar e ‘driblar’ tendências comportamentais inadequadas. A impressão subjetiva relatada é de que o metilfenidato reduz a necessidade de aplicar as estratégias de compensação e os mesmos resultados são obtidos de uma forma mais natural, com menos esforço.

**Conclusões:**

Adultos com TDAH podem ser bem sucedidos socialmente, apesar de sintomas importantes de dificuldades atencionais e disfunção executiva. Eles conseguem ser bem sucedidos por utilizarem estratégias de compensação que demandam vigilância constante. Este pacientes relataram que a necessidade destas estratégias é algo aliviada pelo uso de metilfenidato, embora este possível mecanismo deva ser confirmado em estudos prospectivos de séries maiores de pacientes.

Around two-thirds of children with ADHD carry over symptoms and disability into adolescence and adulthood,^1,2^ with an increased risk for complicated outcomes in a number of psychosocial issues, including low academic and professional achievements, problematic social, familial and marital relationships, and involvement in traffic accidents, alcohol and drug abuse.^2-5^

The impact of ADHD, ranging from a childhood problem affecting performance at school and early parent and peer relationships to a disorder interfering with adult functioning, mirrors the development and engagement of frontal lobe structures in the regulation of social behavior in adults.^6,7^ Recent data have shown a delay in the maturation of cortical structures in children and adolescents with ADHD, most notably in the frontal lobes,^8^ supporting the notion that ADHD adults were at a disadvantage to deal with the massive increases in social pressures characteristic of adolescence and early adulthood.

Interestingly, although as a group adult patients with ADHD suffer the consequences of problematic social functioning, some individuals succeed and function very well academically, professionally and at a family level. Therefore, adults with ADHD are distributed along a functional continuum, with some pertaining to the extremes of this spectrum and many functioning somewhere in between. The author has recently focused studies on ADHD people pertaining to the high functioning end of this spectrum, and particularly how such individuals compensate for their ADHD symptoms. This study presents the case histories of some of these patients, including their experience with stimulant medication. Perhaps these illustrative case reports can serve to kindle the interest in future studies comparing ADHD patients able to be professionally successful with those who are not.

## Patients and methods

From a pool of 63 patients with ADHD diagnosed according to DSM-IV adapted criteria (K-SADS E, version 6.0)^9^ five were selected five who (i) have graduated from university; (ii) are actively working in the field they graduated in; and (iii) are financially independent. Patients were aged between 21 and 55 years of age, and were seen in private practices or at the Behavioral Neurology Clinic of the Hospital São Lucas da PUCRS. Their scores on the Adult ADHD Self-Report Scale (ASRS-18), version 1,^11^ adapted to Portuguese^12^ are shown in Table 1. In addition to probing for ADHD-related symptoms, history taking focused on practical difficulties and on the effortful, time-consuming and other types of behavior used to circumvent these difficulties. The latter were conceptualized as compensatory strategies. In addition, the presence of symptoms of inattention, hyperactivity and impulsiveness during childhood and adolescence, according to the DSM-IV,^10^ were specifically investigated through the K-SADS for childhood symptoms.^9^

**Table 1:** ASRS-18 scores in the Portuguese translation of the scale. Part A refers to 9 symptoms in the domain of inattention, disorganization, difficulties to get activated to start and to conclude tasks. Part B refers to 9 symptoms of hyperactivity and impulsiveness. Symptoms are scored between 0 and 4 points, from absent to very frequently present.^12^

Patient	Part A	Part B	Total
1	34 / 36	26 / 36	60 / 72
2	30 / 36	28 / 36	58 / 72
3	28 / 36	29 / 36	57 / 72
4	22 / 36	20 / 36	42 / 72
5	26 / 36	27 / 36	53 / 36

All patients were taking doses of methylphenidate ranging from 0.7 to 0.85 mg/kg/day. Their subjective views regarding the effect of this medication on their difficulties and impact on their daily life were questioned in an open, unstructured fashion. The latter was seen as relevant because it is not clear what to expect from stimulant medication in high functioning adults with ADHD. In addition, all patients were seen by a psychiatrist, who managed psychiatric co-morbidities, when manifesting. All patients agreed to having their cases reported and published.

## Summary of the case reports

The ADHD-related symptoms reported by these five patients, the strategies they employ in a bid to compensate for their symptoms along with subjective accounts of the effect of methylphenidate on their daily lives are briefly summarized below and in Tables 2 and 3. Complete case reports are contained in the Appendix.

**Table 2:** Main symptoms reported by five successful adult patients with ADHD and the strategies they use to compensate or circumvent these difficulties.

Symptom	Strategy to compensate / circumvent
Difficulties with focusing and persevering on tasks	Active, effortful vigilance to persevere and conclude tasks
Difficulties with reading - do not read or take a long time reading	Development of abilities to grasp the key aspects based upon only partial reading of relevant texts
Taking longer to conclude tasks	Active, effortful vigilance
Tendency to procrastinate	Development of abilities for ‘last minute pushes'
Underestimation of time	
Accelerated forgetfulness	Taking notes all the time / Multiple diaries, alarm clocks, etc
Excessive somnolence	
"Shuffling" priorities	Active, effortful, vigilance to keep priorities
Feeling that could have done better if only...	

**Table 3:** Main subjective effects of methylphenidate reported by successful adults with ADHD.

Reduced need for effortful self-control to attend to the tasks at hand
Increased ability to get started on tasks and activities, thus keeping on schedule
Improvement in the ability to remember meetings and other commitments
Decreased impulsivity, irritability and impatience
Greater clarity to define goals and make decisions

### ADHD- related symptoms

Difficulties with attention and concentration, interfering in a number of activities ranging from reading to attending meetings and even following conversations, were reported by all patients. The group is easily distracted by less relevant stimuli that they encounter. During childhood and adolescence, they all had difficulties concentrating in class and doing homework. As depicted in the case histories (see Appendix) these difficulties either led to their parents being summoned to school or were artificially minimized by either low levels of school demand or high levels of strict discipline at home.

In addition, they constantly struggle with a tendency to procrastinate and ‘shuffle priorities around’. Unless they actively refrain and redirect their efforts, they tend to spend a significant amount of time doing less important, less urgent, and usually more immediately rewarding activities, disregarding deadlines for priority tasks. A related symptom, described in detail by one of the patients, is the need for ‘last minute pushes’ in order to finalize really important assignments that could be done in a much better, more organized fashion should procrastination and postponement not systematically occur. In essence this is ‘running to catch up on lost time’, a term used by many of the patients. At least one patient mentioned she had excessive daytime somnolence, which compounds her difficulties to pay attention and concentrate during the day.

Two other potentially interfering symptoms were reported, namely, memory difficulties and impulsiveness. Forgetfulness was common, but had a peculiar characteristic in which the patients described that they not only tend to forget current appointments, duties and materials, but also tend to forget facts, events and details at a faster rate than their peers. Thus, when discussing what happened or was dealt with during a meeting or a trip, or what somebody said or wrote in a film, a report, a newspaper or a book, they feel embarrassed because they cannot retrieve information that easily comes to mind for other people.

Difficulties with impulse control appeared in different forms. One patient got easily irritated and impatient, and tended to openly argue or got into complicated situations with their clients. Another said things ‘before thinking’ and later regretted that a series of events (in this case, at a building plant) evolved from the decision taken impulsively.

### Compensatory strategies

These five patients have become professionally successful and the strategies they used in a bid to compensate for their ADHD-related symptoms are summarized in Table 2. These strategies could be divided into effort-related and practical strategies. To circumvent the constant drive to distraction, the difficulties focusing for long enough on a given task and the tendency for impulsive attitudes, all mentioned they had to dedicate an enormous amount of energy and control / refrain themselves at a conscious level. As one patient put it, he ‘cannot allow things to ensue naturally’ or else they would not move in the right direction. Others developed the ability – and dispend the energy required – for last minute pushes. These effort-related strategies are felt as tiresome and some patients feel exhausted after struggling to manage their days. Practical strategies of symptom compensation indicated by the patients included taking notes copiously, building complex warning or alarm systems so as not to miss appointments, and reading only parts of texts and then developing the ability to grasp the main meanings from these excerpts. In a sense, these are also energy-consuming.

### Medication effects

A common line in the subjective reports of these patients when questioned in an open, unstructured fashion, was that they felt methylphenidate reduces their need for constant self-vigilance to accomplish daily goals. Participants believed they wasted less time and did not need to invest the constant extra-effort to get things done. The feedback they got from friends, relatives and colleagues is that their impatience, irritability and impulsiveness diminished. In addition, some patients had the impression that their forgetfulness was less marked.

## Discussion

For ontogenetic reasons, the social challenges faced by a child and by an adult are very different and relate to the level of brain development. Thus, the child deals mostly with school and family, whereas the multi-layered universe of the adult includes issues related to academic and professional achievements, a much more complex social network, access to substances such as alcohol and drugs of abuse and, above all, the concept of future. As the prefrontal cortex and its circuitry with other cortical and sub-cortical structures mature, the idea of a future and its links with decision-making in the present becomes a major issue.^6,7^

One of the neurobiological perspectives of ADHD is that of a disorder associated with a delay in cortical maturation, particularly marked in the prefrontal regions.^8^ Therefore, it is understandable that symptoms should reach beyond the constructs of inattention and hyperactivity,^14^ and include elements of a ‘developmental’ executive dysfunction.^14^ This is confirmed by longitudinal studies probing the natural history of ADHD^1-4^ which show that difficulties with school and relationships seen in childhood frequently translate into a high risk adulthood, marked by academic, professional, financial and psychological difficulties. However, population analyses sometimes eclipse the fact that some individuals may eventually evolve into a professionally successful adulthood, in spite of their symptoms. The five patients presented in the present work illustrate this point. Symptoms of these patients, along with their strategies to compensate or circumvent them, and in which ways they feel pharmacological treatment with methylphenidate impacts their daily life shall be discussed below.

## Symptoms of ADHD in adults and possible strategies to compensate

The main symptoms reported by these patients along with the strategies they believe may help to compensate or circumvent difficulties are summarized in Table 2.

All these patients reported a lifelong history of difficulties concentrating, focusing and persevering on tasks and with reading texts. These individuals take much longer to conclude tasks, unless they actively make an extra effort to concentrate for longer periods. They cannot ‘allow things to ensue naturally’ else they will fail. This demands significant dedication and energy. In addition, one patient said she developed the ability to grasp the meaning or the most relevant information of a text from reading just parts of it.

Another set of symptoms concerns activation of behavior and the idea of time, core aspects of the executive functions toward achieving favorable results in the future.^6,7^ The main practical symptom is a tendency to procrastinate, that is, to systematically postpone what the patient knows is his or her priority. A realistic perspective of the passage of time prompts the person to commit only to what he or she will be able to honor, thus avoiding the tension caused by an excess of obligations and the disappointments of missing deadlines. This tendency to procrastinate is a reluctance to engage in activities which (i) are not pleasurable and (ii) demand effort and concentration. Furthermore, it is closely related to the ‘shuffling’ of priorities, in which less important or less urgent (usually also less demanding) matters are taken care of first, relegating more urgent matters for later – often ‘dangerously’ close to deadlines.

One interesting aspect of the executive dysfunction in ADHD is that patients have a reasonably clear idea of their duties and of arrangements with others. However, their acts suggest otherwise, as if they were not ‘anchored’ in the future consequences of such acts. This concept of ‘anchoring’ is relevant: normally, committing to something or someone generates an automatic feeling of obligation and responsibility with the need to honor that commitment. In a sense, the moment we commit, our brain ‘launches an anchor’ to the future and this helps to guide our acts, until we reach the future point where we are anchored, that is, we comply with the obligation we have committed to. In the lack of anchoring, that is, of a brain representation of future scenarios, it is easy to deviate from the trajectory that would lead to the desired consequence.

It should also be stressed that the tendency to procrastinate shown by adolescents and adults with ADHD is not related with the intellectual abilities to actually perform the task. There is, indeed, a very ‘painful’ discrepancy in which on the one hand the person is capable of performing the task, but on the other simply cannot engage to do what has to be done. Other symptoms stem from these difficulties, including the understandable feeling that the task or work could have been done better with higher quality, if only… an adequate amount of time had been allocated to the task. As exemplified in these case histories, some patients try to compensate for this tendency to procrastinate by developing the ability for highly productive ‘last minute pushes’. Even though the latter are often inefficient to avoid the feeling that the final output could have been better, through these pushes at least some documents, texts or presentations are produced, which would not have happened if it were not for this capacity to become focused in the last few hours before deadlines.

Other symptoms depicted in the case histories concern excessive daytime somnolence and memory difficulties. Excessive somnolence is one of the key symptoms of the ADHD-related entity provisionally called sluggish cognitive tempo^15^ and adds to the difficulties in concentrating and getting up top speed, being an easy path to procrastination.

The author has made the (unpublished) observation that a significant percentage of adults complaining of memory difficulties have ADHD, particularly after overt mood or anxiety disorders are excluded. Memory difficulties in patients with ADHD are usually attributed to the pervasive deficit in attention, and as anyone who has attended a lecture or read a paper can attest, retrieving information is indeed more difficult when acquired and stored without the benefit of full attention. However, poor attention may not tell the whole story, and ADHD patients have difficulties keeping information on hold while attending to something else, suggesting a role for impaired working memory. Finally, a mechanism of *accelerated forgetfulness* may be at work. This has been described in patients with epilepsy^16^ and refers to a reduced capacity to access stored information after some period of time. Patients with accelerated forgetfulness have difficulties in recalling facts, experiences and combinations much sooner than their peers, and this has been specifically mentioned by some of the patients. The way to compensate is again labor-intensive: they took notes all the time, keeping multiple diaries, alarm clocks, and often recruiting a network of colleagues and family members to remind them of important meetings and other obligations.

Finally, difficulties with impulse control are a major source of problems for patients with ADHD and reflect poor behavioral inhibition.^3,6^ Impulsiveness crafts a style of decision-making which does not benefit from the use of executive functions. Because of this, socially embarrassing situations commonly derive from precipitated statements or attitudes and also from commitments or promises not anchored in the future – that is, on the real capability of being honored. When a given behavior is no longer adequate, acceptable or useful for the person, it should be inhibited, allowing real time to consider other options and act accordingly. Impulsiveness has to be actively fought and such constant vigilance is indeed exercised by some of the patients as a means of compensation.

Because financial independence is a major social goal, and granting that these patients could be considered professionally successful, they might be regarded as not being functionally impaired, thus challenging the diagnosis of ADHD (10). However, the subjective reports suggest that these patients suffer a great deal to manage their days, and often feel exhausted with and overwhelmed by the extra levels of effort and preoccupation needed to circumvent a tendency for distraction, disorganization and procrastination. Thus, the author proposes that this effortful functioning together with the fact that these patients’ minds are constantly occupied with how to get things done ought to be conceived as a functional impairment. This understanding is also important to interpret their subjective reports on the effects of methylphenidate, as further discussed below.

In a recently published, comprehensive, longitudinal study of a cohort of patients with ADHD, Barkley and colleagues analyzed a large number of symptoms related to executive dysfunction to single out those that best differentiated adults with ADHD from those with other psychiatric disorders and from a healthy control group.^3^ After a series of sophisticated statistical analyses, these authors arrived at nine symptoms including being easily distracted, deciding impulsively, having difficulties to withhold an activity or behavior when this is the most appropriate attitude, beginning projects without adequate planning, not following through on promises or commitments, disrespecting priorities, having a tendency to drive vehicles much faster than other people, having difficulties to sustain attention in demanding tasks, and having difficulties to organize tasks or activities. This evidence provides strong support for the notion that symptoms of executive dysfunction are key features of ADHD in adults. This has significant therapeutic implications, as will be discussed next.

## Therapeutic considerations

Because none of the patients were actively engaged in cognitive behavioral therapy, therapeutic strategy will not be dealt with in the present work. On the other hand, all were taking methylphenidate, four of these for long enough to report on the effects of the medication.

There is a pressing issue concerning stimulant treatment in adults with ADHD because it is not clear how much the medication impacts executive dysfunction which is central to the difficulties of these patients. Well-designed studies on stimulants in adults with ADHD have measured efficacy on the basis of improvement of the core symptoms of inattention and hyperactivity/impulsiveness^17^ while the efficacy for symptoms of executive dysfunction can only be extrapolated. Even more significantly, it is not at all clear to what degree and through what kind of symptom alleviation might stimulants help patients who are functioning well professionally. Thus, it was deemed important to describe the subjective impressions of these patients with the use of methylphenidate (Table 3).

Taken from the spontaneous reports, these patients felt that when using methylphenidate, they were not so overwhelmed while the level of physical and mental effort needed to manage their days was reduced. In other words, they suggested that the medication reduced the need to be ‘en guard’ all the time, which constituted their main effort-related compensatory strategy. In addition, they reported that other people had remarked that medication made them less impatient. Generalizations such as these can only be suggested through case reports, such as those presented here. Of course, these are not scientifically valid conclusions, but are clinical impressions that may help to formulate testable hypothesis. In particular, the different perspective of functional impairment suggested by these reports should encourage the development of instruments to measure quality of life in adult patients with ADHD,^18,19^ which will then allow a more solid view of the impact of medical treatment in these patients to be formed.

## APPENDIX

### Case reports

#### Patient 1

TM is a 34 year-old recently married woman who graduated as a medical doctor at the age of 24. She works as a medical advisor in a drug company and needs to read through the pertinent literature, write reports, prepare presentations, and attend meetings. The quality of her work is high, but she believes she pays a ‘high price’ to reach her level of achievement.

During childhood and adolescence, she was easily distracted at school and her parents were often summoned to discuss her difficulties with attention and concentration. She had no learning difficulties, but could not concentrate for homework. Her parents also complained about poor organization and forgetfulness.

She has difficulties persevering on tasks, and tends to constantly switch focus, being easily distracted by whatever happens around her. In addition, she takes much longer than needed to finalize assignments because she is easily deviated from what she is doing, and tries to do different tasks concomitantly. She compensates by actively and constantly ‘controlling and forcing herself’ with a lot of effort to finish assignments. Because she tends to procrastinate, she is always ‘running’ to make up for lost time, and has the feeling that she could have done things better, if only she could have allocated more time to perform a given task.

She describes herself as a very disorganized person. She is ‘constantly’ losing things, including her diary and tends to miss appointments. The way she tries to compensate for this is to build an alarm system with a number of alarm clocks ringing several times a day.

Finally, she has difficulties remembering where she read a particular text, and keeps trying to remember her sources. She tries to get around this difficulty by writing down everything she believes she might need in the future, thus accumulating a lot of notes.

#### Patient 2

SD is a 48 year-old dentist who has always had problems at school. He could not keep still and concentrate, did his assignments ‘at high speed’, did not comply with rules, and was even expelled from one school. He was hyperactive and had nocturnal enuresis. However, he never failed and was able to graduate and become a technically highly skilled professional.

He feels there is always ‘something’ deviating him from ‘his track’, and he easily ‘gets lost’ unless he puts a lot of effort trying to control his behavior. If he does not control himself, things are simply not done. “They are never done naturally”. He takes a long time getting up to speed in the morning, and delays of up to 2 hours are common before arriving in his office. In addition, he often takes an excessively long time to deal with a given patient, thus disrupting his office schedule.

He says he almost never reads books, because he gets impatient and simply ‘cannot continue’. Because he ‘cannot wait’ he is often regretful for acting impulsively with relatives, colleagues and even with patients. He has had two marriages and is currently separated from his second wife.

Psychiatric evaluation pointed to an anxiety disorder, in addition to ADHD. He has been treated for 4 years with methylphenidate, and believes his abilities to meet his main obligations and get up top speed to start his day and keep on schedule have markedly improved. Furthermore, irritability and impatience have decreased and although he still has difficulties organizing his day, he considers himself ‘a different person’ when on medication.

#### Patient 3

DL is a 33 year-old woman who graduated as a lawyer, has recently separated from her husband and is the mother of a healthy 2 year-old boy. She excelled at an average private primary and high school, but has ‘never’ been able to take notes during her classes, study at home or do homework. School was ‘easy’ and all assignments were done while in class. She got distracted all the time, to the point that her notebooks consisted only of photocopies of her peers’ notebooks. Despite being a competent professional, she has the ‘feeling’ of not ‘having learned anything’ at University.

She runs a practice with two other lawyers and reports difficulties with deadlines, worrying to the point that she ‘dreams’ about missing deadlines. She keeps several ‘diaries’ in different places and in addition asks her colleagues to help her keep track of what she has to do.

She also says she has a lot of difficulties ‘doing first things first’. If she allows her day to ‘flow’, she tends to the produce documents for which she had no deadlines and constantly procrastinates over assignments for which she does have deadlines. She needs to constantly remind herself of her priorities to overcome this tendency. Her procrastination takes different forms, including getting distracted by several other things before sitting down and getting to work. Thus, she got used to working for the whole night to produce documents she could have prepared well in advance. These ‘last minute pushes’ (she considers herself a ‘last minute person’) leads to a feeling that she could have done better, if only she had dedicated more time to any given assignment. Occasionally, however, she fails, when not able to finalize a graduate course on consumer law because she was not able to write the final thesis.

She has always had difficulties reading and memorizing; to read a book has always been a ‘sacrifice’, with a constant need to reread after a page or two.

Her technical reading is peculiar in that she never reads a whole chapter, for instance. She scans the text to the most relevant point or sections, reads these as best as she can, and derives the whole meaning from these few excerpts. Furthermore, she has a very poor recollection of past events, details and conversations.

Oftentimes, when talking, she gets distracted and loses track of what she is saying. During legal proceedings, she needs to keep herself under active control all the time, focusing intensely on a certain point in the hearing, afraid of getting distracted by any surrounding stimuli.

She has been seen by a psychiatrist who found her to be mildly anxious but did not prescribe anti-anxiety medication. She takes methylphenidate and feels she is much less worried with the amount of work she has. Her days flow in an easier, more natural fashion, she pays attention to texts for longer and has a rewarding feeling of ‘getting things done’, that is, she is more ‘centered’ on her priorities.

#### Patient 4

MF is a 49 year-old married woman, and successful architect who has an adolescent son diagnosed with ADHD without co-morbidities. Performance at school was marked by difficulties with attention and concentration, which were eclipsed by the strict discipline she experienced at home, which left ‘no room for failure’.

She is easily distracted, has difficulties with reading, and often cannot keep focused on her priorities. In addition, she complains of memory problems which interfere with her ability to remember details of discussions, films or news after only a few days, and needs to be constantly reminded of appointments. Furthermore, daytime somnolence has been a problem for decades.

She is well regarded in her field, has clearly succeeded, but believes has always paid a ‘high price’. She needs to discipline herself in a very effortful fashion just to get through her daily activities. She was seen by a psychiatrist who ruled out major mood or anxiety disorders.

She was started on methylphenidate and after adjusting the dosage she believes her thoughts flow with much more objectivity; she finds it easier to construct a line of thought and pursue specific results. In addition, she states she has been able to work more productively, get more things done and her daily somnolence has markedly diminished. On the other hand, she has been tackling insomnia and transient palpitations.

#### Patient 5

SAD is a 53 year-old architect, married, with two children who are finishing University. She always had difficulties at school, caused by her lack of attention, concentration and tendency to procrastinate on her school assignments. She started at a public and later moved to a private primary school, but failed fourth grade, and then failed again during high school. She had difficulties gaining admission to University and also failed some disciplines, until finally graduating.

She reports increasing difficulties with memory; she cannot retrieve what she reads or hears. In addition, she has been having difficulties verbalizing her ideas, as if she could not find the words to express what is in her mind. The ideas cross her mind faster than she can express them, as if they ‘departed from her head’.

She was seen by a psychiatrist who diagnosed mild depression, and has a past history of a typical episode of transient global amnesia at age 48. A complete neurological investigation at the time, including EEG, MRI, carotid and heart ultrasound were normal.

Some of the practical difficulties she encounters include forgetting to pose relevant questions, the answers to which are crucial to move forward with her projects. Often, after a meeting, she *remembers she forgot* to ask that simple question which had crossed her mind, but simply disappeared from consciousness until it was too late.

She was invited to write down some other aspects, thoughts and episodes of her life. Some of these are reproduced verbatim below:

“ ... I had to develop mechanisms so that, during a conversation, people do not perceive that, in order to retain any given details of the conversation, I have to concentrate with much effort. Otherwise, in a flash, the whole thing disappears from my mind. I cannot verbalize at the same pace I think, I get distracted with other ideas and start to stutter and then say nothing… which frustrates me lot.”

“At work I have become very careful and have learned to describe all arrangements in a report, before giving instructions to the workers on a construction site. Even taking these careful steps, the workers sometimes do things differently than they should, and say I had told them to do so. I have to take notes of everything I ask, so as not to lose my authority in the field. In summary, my professional life is very effortful. To minimize flaws I have to go to the construction sites everyday, which would not be needed if I could get better organized and efficient. In addition, I review the projects ‘n’ times before concluding, afraid of major flaws. I often leave home but have to come back after a block or two because it crosses my mind I forgot something. When I am home, I start to do something, but then, in the middle of this activity I start to do something else, and then lose track of what I was doing in the first place. Thus, I start wandering around the house trying to remember what it I was I had to do.”

“People complain that I keep repeating the same question several times. I have the impression that I get distracted, and do not really listen to the answer.”

She was started on methylphenidate, and in the 12 months since taking this medication says she functions more at ease at work and at home. It is easier to write proposals related to her projects and the day in the office needs ‘less internal and external control’. In addition, she believes she is being more effective and not as forgetful in her daily activities. She can start and finish assignments. The contrast with not taking medication is ‘dramatic’. She interrupted treatment for 3 months and again had a hard time ‘getting up to speed’ to do what had to be done, and resumed the ‘routine’ of constantly losing objects and forgetting activities. Besides focusing better and thus forgetting less, she believes the avoidance of procrastination is the most useful effect of the medication.
